# Tracking health seeking behavior during an Ebola outbreak via mobile phones and SMS

**DOI:** 10.1038/s41746-018-0055-z

**Published:** 2018-10-02

**Authors:** Shuo Feng, Karen A. Grépin, Rumi Chunara

**Affiliations:** 1 University of Waterloo, Department of Statistics and Actuarial Sciences, Waterloo, Canada; 2 Wilfrid Laurier University, Department of Health Sciences, Waterloo, Canada; 3 New York University, College of Global Public Health, New York, USA; 4 New York University, Tandon School of Engineering, Computer Science & Engineering, Brooklyn, USA

**Keywords:** Developing world, Health services, Public health

## Abstract

The recent Ebola outbreak in West Africa was an exemplar for the need to rapidly measure population-level health-seeking behaviors, in order to understand healthcare utilization during emergency situations. Taking advantage of the high prevalence of mobile phones, we deployed a national SMS-poll and collected data about individual-level health and health-seeking behavior throughout the outbreak from 6694 individuals from March to June 2015 in Liberia. Using propensity score matching to generate balanced subsamples, we compared outcomes in our survey to those from a recent household survey (the 2013 Liberian Demographic Health Survey). We found that the matched subgroups had similar patterns of delivery location in aggregate, and utilizing data on the date of birth, we were able to show that facility-based deliveries were significantly decreased during, compared to after the outbreak (*p* < 0.05) consistent with findings from retrospective studies using healthcare-based data. Directly assessing behaviors from individuals via SMS also enabled the measurement of public and private sector facility utilization separately, which has been a challenge in other studies in countries including Liberia which rely mainly on government sources of data. In doing so, our data suggest that public facility-based deliveries returned to baseline values after the outbreak. Thus, we demonstrate that with the appropriate methodological approach to account for different population denominators, data sourced via mobile tools such as SMS polling could serve as an important low-cost complement to existing data collection strategies especially in situations where higher-frequency data than can be feasibly obtained through surveys is useful.

## Introduction

As the recent Ebola virus disease (EVD) outbreak in West Africa highlighted, collecting data on health and health seeking behavior in places with an existing weak health system using traditional public health sources, especially in the midst of an acute public health emergency or other humanitarian crises, can be challenging or impossible. During the EVD outbreak, routine data systems were dismantled in heavily affected countries.^1^ Disintegration of health care systems can have a profound impact during emergency situations, as clinics become overwhelmed or nonfunctional.^2^ An infectious disease outbreak can thus catalyze further health issues in a community as communities lose trust in the healthcare institutions available and decrease healthcare system utilization.^2,3^ In particular during this EVD outbreak, there was a perceived compromise in maternal health care services as health workers followed the EVD “notouch” policy,^4^ there was scarcity of personal protection equipment,^5^ pervasive community perceptions of a higher risk of contracting EVD at health care facilities,^6^ and a general lack of trust in government services.^7^ Furthermore, health care workers who feared being infected with EVD while working abandoned health facilities, which in many cases were already understaffed.^7^ It is therefore also important to study the indirect health effects of health system breakdown, which should be examined at the population-level and throughout the recovery phase. In particular for maternal health in Liberia, with the closure of healthcare facilities and the loss of health workers (two major health system building blocks) there was a risk of reversing important gains this country had made in the lead up to the outbreak in maternal and newborn health.^1^ Finally, it is also important to understand the mechanisms behind any health system deterioration to mitigate future challenges.

Studies of maternal health outcomes in Liberia during the EVD outbreak have concluded that there were severe decreases in the number of pregnant women who accessed antenatal care during, compared with before the EVD outbreak.^1,8,9^ The reported number of facility-based deliveries also declined drastically during the EVD outbreak compared with pre-EVD levels.^9,10^ This work has, however, described certain limitations in the studies due to the data used. Specifically, some studies used routine health information system data (e.g., the Liberia District Health Information Software) which mainly represents public-sector institutions, while other work using surveys assessed facility-based deliveries without explicitly distinguishing public and private institution use.^1,10^ However, there exist numerous for-profit, religious, and not-for-profit clinics operating in Liberia, especially in urban areas like Montserrado County, which do not report to the Ministry of Health and thus would not be captured in health information system data.^10^ It is also reported that there is no knowledge of the quality of this health information data over time pre- and post-EVD, which was likely adversely affected by the health system disruption during the outbreak.^8,10^ Though other studies have used community-based household surveys to obtain data outside the reporting system, surveys have been limited in their reach; only focusing on specific places in Liberia such as River Cess, Margibi and Bong counties or the city of Monrovia. There was not a survey that was national in scale, and thus each individual survey is not likely to represent regions with different epidemic burdens.^8,9,11^ In neighboring Sierra Leone, a study showed that the decline in number of deliveries and caesarean sections shortly after the onset of the EVD outbreak was mainly attributable to the closing of private not-for-profit hospitals rather than government facilities.^12^ Notably, to perform that study and reach all types of facilities, community health officers collected weekly data from all types of facilities, but difficulties in data collection in the midst of an epidemic were highlighted. In sum, existing studies indicate that new methods to rapidly collect and assess data on measures of health and health seeking behavior at scale, in such acute situations, are needed.

The potential of mobile phones to provide new platforms to collect population-level health data has often been cited for a number of reasons. Population-based surveys, such as household or health facility surveys, can take months to deploy, and it can be months before such data are available for analysis.^13^ Thus, in some scenarios, many measures of the overall population are out of date or simply unavailable, if they cannot be measured by traditional survey mechanisms (such as due to recall issues). On the other hand, the use of mobile phones can be more cost-effective than traditional data collection methods, they can be used to collect data that is difficult to obtain in places with low levels of healthcare infrastructure, they offer opportunity for data collection at high temporal/spatial resolution, and finally self-reports on mobile phones can suffer less from recall and information biases.^13,14^ However, while mobile phone utilization has increased rapidly around the world, there are open questions about how the data can be used, given that mobile phone users are not likely representative of the general population. Methods for using such observational data are continuously improving in their ability to generate accurate knowledge of the population-at-risk (denominator), which is a fundamental requirement in public health for measuring effects and gauging impact.^13,15^ Challenges in assessing the denominator have even been deliberated for traditional healthcare-based data sources.^16^ To adjust the distribution of responses from mobile-collected data, previous studies have employed a method known as raking, which weights responses to census data using demographic variables such as age, gender, education, or geographic location (e.g., urban or rural) to generate representative samples.^17^ Weighting across demographic variables, however, can be highly sensitive to sample size, and results can be confounded by the factors used as weights.^18^ In addition, because household survey data is representative of only the time period in which it is collected, unless census data are repeated regularly, the weights used might be outdated.^19^ For example in Liberia, the last national census was conducted in 2008, 6 years prior to the EVD outbreak, which given the rapidly changing population dynamics in Liberia, including dramatic changes in fertility, rural to urban migration, and other post-civil war changes, such data was also likely unrepresentative in other ways. Furthermore, census data does not usually include health outcome information and surveys which do are based on their own population-at-risk which may not be representative of the entire population either. Thus, mobile phone data has the potential to provide high-resolution windows into public health phenomena especially during an acute situation like the EVD outbreak, though it is necessary to investigate how we can draw inferences from mobile phone survey data, and assess comparable outcome information knowing that the populations captured by mobile phone or other surveys are likely to differ.

To accomplish the above, we employ adjustment via propensity score matching; a method that is commonly used to draw inferences from observational data sources.^15,20,21^ We use this approach in order to compare outcomes measured from an SMS-based mobile survey during the EVD outbreak, to data collected from traditional household surveys. Overall, using mobile tools enabled rapid deployment and collection of real-time, high frequency data immediately following the Ebola outbreak in Liberia at a time when other forms of data collection were challenging. We were able to measure a significant change in facility-based deliveries during the EVD outbreak relative to after the outbreak, in Liberia. We also show that after the outbreak, the proportion delivering in public institutions returned to baseline levels that were consistent with data collected from the most recent Demographic and Health Survey (DHS) (Table 2). Using data from SMS polling of individuals also enabled us to capture information regarding the use of different types of facilities (e.g., private versus public), as well as garner data across all counties in Liberia in near real-time. The lessons from our study in Liberia, we believe, are likely to be useful in many contexts using mobile phone data to collect data complimentary to surveys at scale and at high frequency, during regular or acute emergency situations.

## Results

### Data demographics

SMS survey data were obtained via GeoPoll (GP) (details described in Methods section) using quota sampling to ensure that the distribution of the SMS data was geographically representative of the Liberian population and consisted of a 50/50 gender ratio of respondents, both which correspond to the latest, although outdated, census estimates.^22^ The overall number of completed SMS surveys ranged from 2.74 per 1000 population in Montserrado, the most populous county in Liberia, to 3.75 per 1000 population in Grand Kru, the least populous county; SMS surveys were representative across urban and rural counties.

The overall final sample of respondents was young (30.2 ± 9.1 years) and well educated (44.6% with secondary and 33.6% with post-secondary level of schooling). We found that while our sample of respondents had a similar age patterns as the most recent census,^23^ the very young are not captured in the SMS survey due to constraints on the minimum age (15 years) that was implemented due to ethical considerations and consistent with the minimum age in the DHS survey.

### Propensity matching

To assess types of locations for deliveries, we restricted the GP sample to those who reported a recent birth (12 months prior), and outcomes were measured by balancing the GP population with results from the latest Demographic Health Survey in Liberia. We compared the types of facility used for those who reported births before the outbreak versus those who reported births after. In assessing successive time points to divide the outbreak and “post” outbreak times, January is where we see a rise in the institutional-facility deliveries (significant difference considering January as after the outbreak, compared to months “during” the outbreak, *χ*^2^(1) = 5.40, p = 0.020), opposed to using December as after the outbreak (*χ*^2^(1) = 2.19, *p* = 0.139). We continue to see this difference in February (*χ*^2^(1) = 3.99, *p* = 0.046) and March (*χ*^2^(1) = 6.19, *p* = 0.011). Additionally, our data indicates the proportion of births in public facilities starts to increase around the same time in January, and following this month even showing no significant difference in the proportion of births in public facilities between DHS and GP (*χ*^2^(1) = 4.91, *p* = 0.027 in December, *χ*^2^(1) = 3.91, *p* = 0.048 in January, *χ*^2^(1) = 2.14, *p* = 0.143 in February and *χ*^2^(1) = 2.54, *p* = 0.111 in March), (Table 2). Both of these findings indicating a change in delivery-practices that are evident starting in January. This pattern over time is also confirmed by retrospective studies using healthcare data which showed a significant lowering in the number of EVD cases by January 2015.^1^

**Fig. 1:**
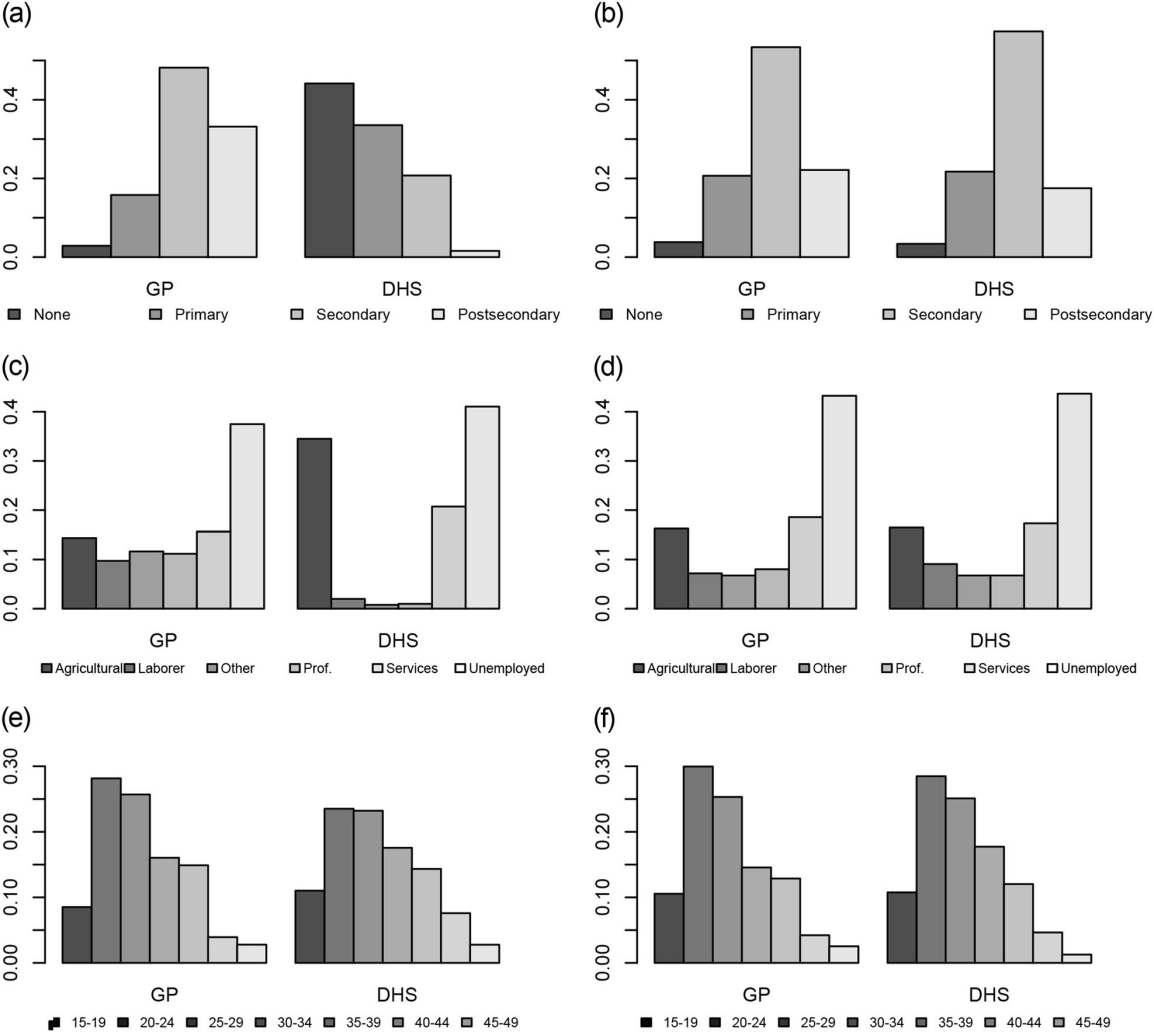
Survey responses by demographics (education level, occupation, and age) prior to the match (**a**, **c**, and **e**, respectively) and after the match (**b**, **d**, and **f**)

**Fig. 2:**
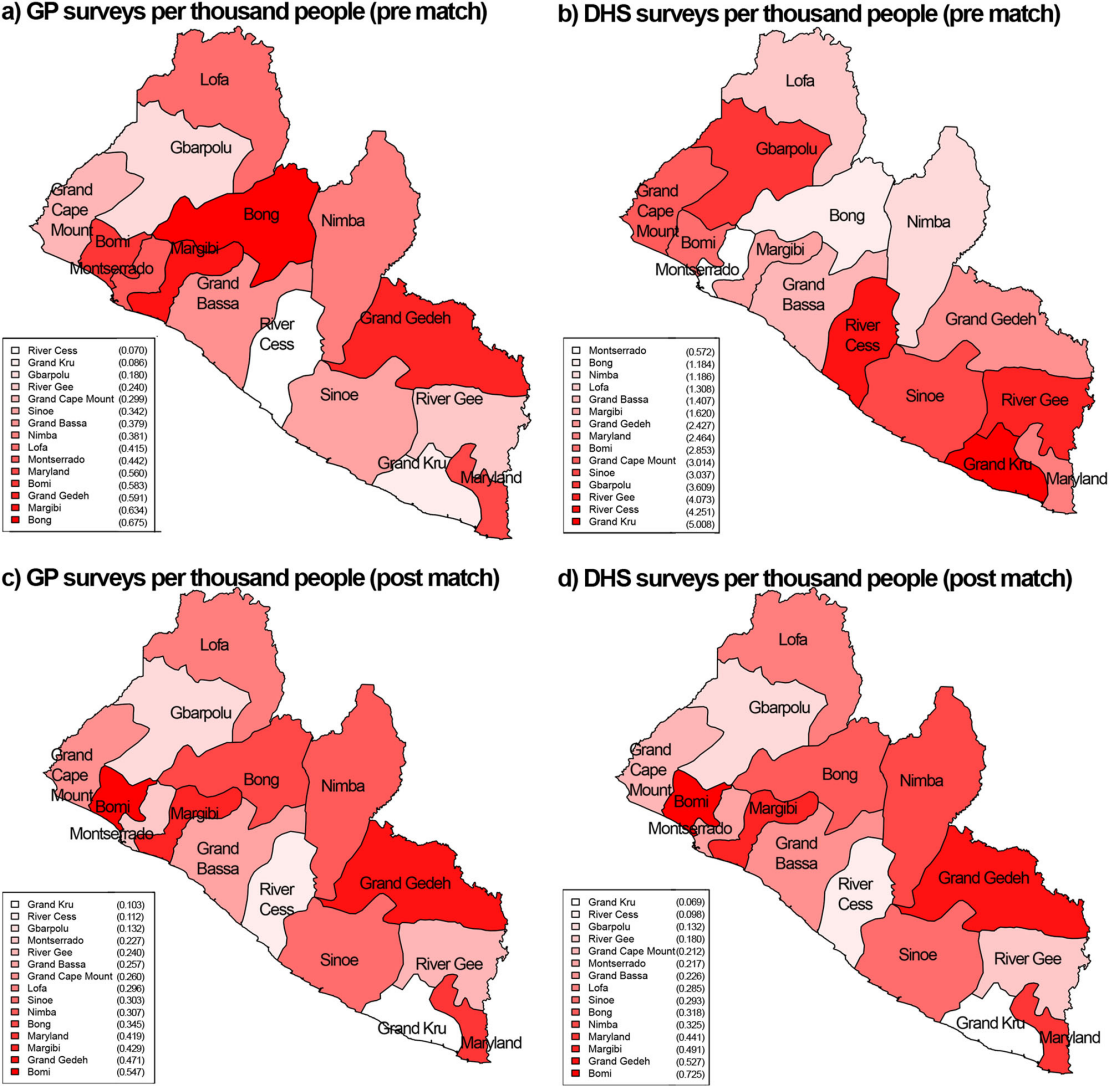
Map of survey responses per 1000 capita by county in Liberia, prior to the match **a** and **b** and after the match **c** and **d**

The distribution of demographic and other variables from the overall GP population, the subset used for comparison and DHS data are described in Table 1. In comparing the GP and DHS populations, they had a similar mean age, though the GP population age range was wider. Both the GP and DHS populations were most represented in the top two counties in Liberia in terms of population (Montserrado and Nimba). The GP population was more educated, with most individuals having completed secondary or post-secondary levels of education while in the DHS most individuals had completed no schooling or primary school. In both groups there was a large proportion of individuals who reported being unemployed (41.6% in GP and 41.1% in DHS). Also, there were more individuals who reported being in labor or technical occupations in GP. After matching, the populations became very aligned on each of these attributes. Regarding geographical distributions, prior to the match the GP surveys were focused in the more urban areas of the country (highest number of surveys per thousand people in the counties near Montserrado county, Fig. 2a). The DHS surveys on the other hand, had the highest number of surveys per thousand people in the opposite, more rural counties of the country (Fig. 2b). After matching, both the GP and DHS populations were similarly distributed across the country (Figs. 2c, d). Figures 1 and 2 illustrate the full distributions across age, occupation, education and location, and standardized mean differences between groups are included in Supplementary Table 3a,b and propensity scores illustrated in Supplementary Figure 1.

**Table 1:** Distribution of GeoPoll SMS responses for the entire population, and population from GeoPoll and DHS (Demographic Health Survey) included in the matching analysis

	Overall Responders (*n* = 5900) *N* (%)/mean ± SD	Included in births analysis (*n* = 1540)	DHS included in analysis (*n* = 5337)
Response dates	6 March–11 June 2015	6 March–11 June 2015	10 March–19 July 2013
Mean age (years)	30.2 ± 9.1	29.8 ± 8.8	28.7 ± 7.7
Min age (years)	18	18	18
Max age (years)	92	49	49
Responses by county (% of total)			
Bomi	164 (2.8)	49 (3.2)	240 (4.5)
Bong	719 (12.2)	225 (14.6)	395 (7.4)
Gbarpolu	60 (1.0)	15 (0.1)	301 (5.6)
Grand Bassa	326 (5.5)	84 (5.5)	312 (5.8)
Grand Cape Mount	132 (2.2)	38 (2.5)	383 (7.2)
Grand Gedeh	240 (4.1)	74 (4.8)	304 (5.7)
Grand Kru	20 (0.3)	5 (3.2)	290 (5.4)
Lofa	405 (6.8)	115 (7.5)	362 (6.8)
Margibi	519 (8.8)	133 (8.6)	340 (6.4)
Maryland	266 (4.5)	76 (4.9)	335 (6.3)
Montserrado	2277 (38.6)	494 (32.1)	640 (12.0)
Nimba	591 (10.0)	176 (11.4)	548 (10.3)
River Cess	16 (0.3)	5 (0.3)	304 (5.7)
River Gee	58 (1.0)	16 (1.0)	272 (5.1)
Sinoe	107 (1.8)	35 (2.3)	311 (5.8)
Education (% of total)			
No school	131 (2.2)	29 (2.0)	2356 (44.1)
Primary school	813 (13.8)	230 (15.0)	1790 (33.5)
Secondary school	2632 (44.6)	713 (46.3)	1107 (20.7)
Post-secondary school	2324 (39.4)	568 (36.9)	84 (1.6)
Occupation (% of total)			
Agriculture	636 (10.8)	224 (14.5)	1841 (34.5)
Laborer	520 (8.8)	141 (9.2)	106 (2.0)
Professional or technical	906 (15.4)	187 (12.1)	52 (1.0)
Sales and services	700 (11.9)	195 (12.7)	1106 (20.7)
Unemployed	2457 (41.6)	633 (41.1)	2191 (41.1)
Other	681 (11.5)	160 (10.4)	41 (0.8)

### Birth location results

In the unmatched samples, the GP population reports most births in public locations, followed by private and then home/other. On the other hand, the DHS population reports births in home/other almost as much as in public locations, and a much smaller proportion in private locations. Within the GP data, participants reporting a birth in a public facility increased after the outbreak by 6.3%. Deliveries in private facilities decreased (by 3.3%) and deliveries in home or other locations (i.e., non-institutional) decreased, although not significantly (Table 2). Comparing the matched population responses for those with babies born before and after the outbreak, the proportion reporting deliveries in a public facility still increased (only 2.2%, but still not significantly different from the DHS data. The proportion reporting a delivery in a private location now increased, and overall the shift back to institutional births after the outbreak was significant (*p* < 0.05).

**Table 2:** Percent reporting birth in different locations during and after the outbreak (unmatched and matched samples)

	Birth Location	GP—during outbreak	GP—post outbreak	DHS - (values matched with GP during outbreak, when matched)	DHS - (values matched with GP post outbreak, when matched)
Unmatched	Public	44.0	50.3^†^	47.6	47.6
	Private	37.3	34.0	8.1	8.1
	Home or Other	18.7	15.7^*^	44.4	44.4
Matched (Decemeber 2014 post)	Public	43.7	48.4	49.6	55.9
	Private	36.0	34.9	16.5	18.5
	Home or Other	20.3	16.7	33.9	25.7
Matched (January 2015 post)	Public	44.3	46.5	52.5	53.0
	Private	34.6	36.1	17.3	18.3
	Home or Other	21.1	17.4^*^	30.2	28.7
Matched (February 2015 post)	Public	44.6	46.3^†^	53.5	51.9
	Private	35.1	38.6	14.8	18.7
	Home or Other	20.3	15.1^*^	31.7	29.4
Matched (March 2015 post)	Public	43.6	47.9^†^	52.3	54.3
	Private	35.1	37.5	15.3	17.8
	Home or Other	21.3	14.6^*^	32.5	27.9

DHS values are from 2013 (thus unmatched values are the same for the during/post groups, matched represent DHS (Demographic and Health Survey) values matched with GP (GeoPoll) values from during/post outbreak)

^*^p < 0.05 between GP during and post-outbreak data

^†^no significant difference between GP and DHS value

## Discussion

In this study we demonstrated the use of mobile phone surveys via SMS for gathering information about health seeking behavior data during and in the immediate aftermath of an EVD outbreak; critically important for understanding the collateral effects from the EVD outbreak on public-sector primary healthcare delivery which are conjectured to greatly exceed the direct effects of EVD infections.^10^ A pressing concern with the use of many Internet and mobile-enabled tools, such as SMS, is that the population denominator is not representative of the general population. We investigated the population that was reached using this methodology and used propensity score matching, which is commonly used in the statistical analysis of observational data, to create balanced populations for estimating and comparing outcomes regarding birth location and accounting for the covariates that predict being in the SMS population group compared to a DHS survey in Liberia. We demonstrate that with appropriate methodological considerations, it was possible to generate matched groups with survey (DHS) data, across which to compare outcomes. Before matching, there was a greater proportion of deliveries in public or private facilities in the GP population, compared to the DHS population, which makes sense given the demographics of the GP versus DHS populations (more educated and a higher proportion in professional/technical occupations, indicating a higher socio-economic status). Once matched, we found that matched subgroups had similar patterns of health facility delivery location in aggregate (higher proportion of private-facility deliveries in the GP matched population than the DHS), and utilizing data on the date of birth, we showed that deliveries in institutional-facilities were significantly decreased during, compared to after the outbreak (*p* < 0.05), whereas in the unmatched population the proportion of deliveries in a private facility decreased post-outbreak. Directly assessing behaviors from individuals via SMS also enabled the measurement of public and private sector facility utilization separately; our data also suggest that use of public facility deliveries returned to baseline values after the outbreak. While our analysis was retrospective, data from mobile tools can be captured in real-time, and the conclusions of this paper indicate that prospective SMS surveying and analysis can offer a great opportunity to understand such disruptions and to inform approaches to mitigate such potential risks in real-time.

Results confirmed what has since been found in many retrospective studies of healthcare utilization in the outbreak.^1,8–10^ After matching, we found that there was a significant change in facility-based deliveries during, compared to after the outbreak. As our approach enabled gathering information about the types of facilities used (public versus private) across the nation, in our data, this change was due to a similar lowering of births happening in public facilities and private facilities. A previous study of birth locations found that the decrease was mostly due to decline in deliveries in public locations, but the study was limited to just Monrovia^11^ which is where the highest proportion of private facilities are located in Liberia. Our finding is in contrast to what was found in Sierra Leone, where the decline in number of deliveries in the EVD outbreak was mainly attributable to the closing of private not-for-profit hospitals, rather than government facilities.^12^ After the outbreak, the proportion delivering in public institutions as measured through SMS returned to the baseline (DHS) levels, and the proportion delivering in private locations also increased.

The matching approach used here could also be applied to other observational data sources. For example, GP provides a range of mobile polling options ranging from SMS to Interactive Voice Response (IVR). IVR is sometimes believed to be more effective in situations in which there is a low rate of literacy, but is more costly to administer.^24^ While some studies have examined the possibility of mobile surveys via IVR, we add to this literature by conducting an SMS-only study at scale. Existing work has reported attrition related to length, so IVR might be worse than SMS in some respects,^17^ it is hard to exactly make any comparison between the methods as SMS is designed by nature to be a shorter medium. Since there is less work using SMS at similar scales for health surveillance and we elected to test SMS polling in this context.

In any adjustment and matching exercise, there are inherent assumptions that must be recognized. The assumption of ignorability of unobservables is imposed by this inference method; in other words, we are assuming there are no unmeasured variables which confound the relationship between the treatment (surveyed via GP) and the outcome (delivery location type).^25^ Practically, we have attempted to ensure that covariates included do not depend on the outcome variable, introduce bias or have some tautological relationship with the outcome variables examined. While we cannot verify it, the ignorability assumption is generally assumed reasonable because matching on or controlling for the observed covariates also matches on or controls for the unobserved covariates, in so much as they are correlated with those that are observed. Further, we do recognize that unmeasured covariates can exist. We perform our study with this awareness, and with the attempt to include covariates that would describe general demographics, and that were feasible to measure using the SMS survey. There can also be unmeasured covariates due to inherent limitations of the data source. First, simply through human error there can be issues in who is reached (for example, the quota sampling for gender was not accurately implemented and in the first round of the survey only 40% of respondents were female). As with any self-reporting, there can be limitations based on the accuracy of the data; however given that we found most reports from the same User ID had consistent demographics reported (and we discarded data from any User IDs with inconsistent attributes), we have confidence in the validity of data used. There may also be biases caused by external factors that may result in a correlation between which type of healthcare facility is used and who is surveyed via GP that is spurious, not causal. Phrased differently, it is possible that people who are surveyed through GP are more likely to give birth in certain types of locations (e.g., if types of facilities available differ by location, or the telecommunications provider that GP uses represents only one group from the population—we would never be able to adjust for the unrepresented groups). Broadly, from any mobile-phone data and also as illustrated in the summary of data pre-match from GP (Table 1), we recognize that the SMS data comes from a generally more young and urban population than DHS. As described, we use the analytic methods (propensity matching), to adjust for this given that the DHS survey (amongst other surveys) are also not representative of the population. In the future, mobile phone numbers and consent could be garnered during household surveys, which can then be used for longitudinal data monitoring of a sample drawn from a known sample frame.

To improve this work, as in any effort to decrease the amount of confounding, it would be very pertinent to increase the number of covariates. This could be done by ascertaining more baseline demographic information during the survey process, even without specifically knowing prospectively what population will be reached. Future research should involve replicating this work in other situations and places, improving the scale of data collection to improve statistical significance as well as improving data matching methods with further covariates and outcomes. Additionally, the work is limited by the types of data that are available for comparison. Though we used county-level resolution for comparability with the DHS-measured outcomes, it is possible to obtain even higher geographic resolution via mobile tools. As well, “gold standard” data sources used here are outdated as the DHS data is three to four years old. We thus see mobile data collection as a useful complement to data on health and health seeking behavior collected from household surveys as demonstrated here, particularly for more nimbly capturing changes in health-seeking behaviors that traditional surveys allow. As mobile phone ownership, and even smartphone use is growing even among poorer segments of the population, and given its low cost, with appropriate methodological approaches, it can be a valuable tool for population health intelligence.

## Methods

### GP data

To study health-seeking behavior in Liberia, we designed a survey which covered several topics, such as if the respondent had direct knowledge of anyone who was sick with Ebola, and type of delivery location, if a recent birth was reported. Maternal health services, which are dependent on functioning health systems, are likely to be particularly susceptible to external shocks, such as an EVD outbreak, and delivery locations are important for tracking health seeking behavior. Thus analysis of responses to the survey question regarding delivery location is the focus of this study.^9^ We commissioned GP (www.geopoll.com, GP) to deploy the survey. GP is a commercial SMS survey firm with existing operations in Liberia. GP works with mobile cell operators to collect a list of recently active cell phone users in the countries where it operates. In Liberia, it works with the cell phone provider the MTN group, which is the leading provider in Liberia.^26^

Quota sampling was used to recruit a sample of respondents that was reflective of the geographic distribution of the Liberian population at the county level (based on data from the 2008 census). As well, a population at least 50% female was targeted. To achieve these quotas, GP targeted surveys to respondents until the desired proportion of completed surveys (by location and gender) were achieved. Survey respondents were provided $0.50 USD in phone credit as an incentive for correctly completing the survey (and use of compensation has been shown to increase participation via mobile surveys).^17^ Full costs for the survey are reported in the Supplementary Information. Between March and June 2015, we conducted 4 rounds of surveys (roughly 1 month apart). Each round returned exactly 2500 completed surveys for a total of 10,000 completed responses, 6694 of which were from unique respondents. Table S1 describes full details of the survey response rates and time periods. Overall, the GP demographics are indicative of a generally young and more educated population, while the DHS population is only women within a certain age range with less professional occupations which indicates that each survey has its own underlying population, and populations even within surveys must be matched more carefully before comparisons can be made.

Among other questions, which mainly focused on awareness of Ebola and recent health seeking behavior, respondents were also asked to provide information on any recent health and health seeking behavior including whether or not there was a recent birth in the household and who the respondents thought who should be responsible for health service delivery in their communities. A list of the exact questions used in this study is provided in the Supplementary Information file. The survey also asked respondents to provide demographic and socio-economic details including occupational status and education. GP provided us data on age and geographic residence of respondents. For the matching analysis, we selected only those who reported a birth, anywhere in the country, within the past 12 months of the survey date. Full details of the entire sample and those included in the analysis are described in Table 1.

### SMS data validation

To examine data quality and reliability, we used repeat responses from the same respondents in the survey. As the SMS data were collected at four different time points (2500 surveys per time point (Supplementary Information Table 1)), a portion of our respondents answered the survey more than once, and we examined consistency in those data to evaluate data quality. Of the 10,000 total responses, 6694 were from unique responders (each responding phone number is denoted by a unique User Id from GP). Of these responders, 2220 answered the survey at more than one time point (2, 3, and 4 being the possible number of surveys from any individual responder). For the most part, responses from repeat responders were consistent across age, gender and region; 794 had at least one discrepancy in age, gender, and region. This could, for instance, be due to sharing of phones which can be common.^27^ In these cases (when there was a discrepancy in any response), all reports from that User Id were excluded from all analyses.

### Demographic and Health Survey Data (DHS)

DHS are nationally-representative household surveys of reproductive aged women (in some countries, males are also surveyed in a proportion of eligible households) that provide data for a wide range of population, health, and nutrition measures.^28^ The data are recoded by different groupings including by households, eligible women, and eligible men and it captures information about reproductive age women ages 15–49. Survey questions are largely standardized across countries and most countries repeat the studies only about every 5 years. The DHS use a two-stage sampling strategy in which a random sample of geographic areas (usually enumeration areas from the most recent national census) and then within selected geographic areas households are randomly selected for inclusion. DHS are widely considered the most rich health surveys conducted in developing countries.^29^ The most recent DHS was conducted in Liberia in 2013.^30^ The location of births was asked to women in the study, and ages ranged from 15–49. We included data from all those in the 2013 survey who reported a birth, anywhere in the country, and full details of the sample are described in Table 1.

### Propensity score matching analysis

The ultimate goal of propensity score methods is to reduce the selection biases in analyses involving data in which the outcome may be confounded by underlying attributes of the data. In the case of this study, participants who give birth at different types of healthcare facilities presumably do not do so at random. There is theoretically an underlying attribute (demographic) trait or traits that relates to what type of location is used. Matching participants using propensity scores offers the ability to at least partially eliminate such biases. While we match the GP and DHS populations here, the DHS survey is only performed every few years. We thus use the nimble opportunity of SMS surveys (which can be deployed more frequently) to assess outcomes during and after the EVD outbreak (the DHS data doesn’t change). We summarize the types of facilities for deliveries for the original unmatched GP population, and then GP population matched to the DHS during and after the outbreak.

The propensity score is a probabilistic metric, which is calculated for each individual in a study based on observed baseline characteristics. In our study, the propensity score is the predicted probability of an individual being assigned to a particular “treatment” (here, being captured in the GP versus DHS survey) given a set of observed covariates. Balancing populations via the propensity score aims to reach balance between the treated and control groups as best as possible, instead of trying to weight one like the other—as both encompass different populations. Then, outcomes (in this case, delivery location) can be compared based on these matched populations. The first step of propensity score matching is to estimate a propensity score for each case. To accomplish this, a logit regression is performed with the survey type (defined dichotomously) as the dependent variable and all available demographic variables as its predictors. We adjusted for these potential confounding factors using a regression model with each factor as a covariate: age (represented as a continuous variable), education (categories: none, up to primary, up to secondary, up to post-secondary) and occupation (categories: unemployed, sales and services, agriculture, professional or laborer).^31^ We chose covariates for matching which are recognized to represent basic demographics and were available from our data sets (age, education level and occupation).

We separated the GP data by those who reported a birth during the outbreak and after the outbreak. We analyzed different times for dividing “during” and “post” outbreak (“during”: the birth was between March 2014 and either November 2014, December 2014, January 2015, February 2015 or March 2015, inclusive), and after (including births after the “pre” time period until June 2015). Once the propensity scores are calculated, there are multiple matching methods that can be used. We used a standard caliper which requires that only matches within a certain caliper are acceptable and thus units without an acceptable match are removed. We matched the DHS group with the outbreak group from GP and the post-outbreak group. After matching, we compare the outcomes of these adjusted groups (location of delivery: public, private or home/other facility). The maximum permitted difference between matched subjects (the “caliper” used) was 0.2.^32^ The standardized mean difference between matched groups (GP and DHS) for each category were under 0.2 (except for age post-outbreak which was slightly higher due to the decreased number of observations), indicating sufficient balance.^33^ Differences in proportions of deliveries in a given location-type during compared to after the outbreak, or between GP and DHS data, are assessed using a *χ*^2^statistic to test for equality of the proportions (two-sided).

### Ethical approval

This study protocol was reviewed and approved by the NYU University Committee on Activities Involving Human Subjects. Since GP conducts surveys through the mobile phone, consent was conducted through SMS or voice messaging (as compared to traditional paper methods).

## Electronic supplementary material

Supplementary information

Supplemental Figure 1

## Data Availability

Anonymized data used in this study are available at the Harvard Dataverse: 10.7910/DVN/W4KBB4. DHS data is available for free after a registration process: https://dhsprogram.com/Data/.

## Electronic supplementary material

**Supplementary information** accompanies the paper on the *npj Digital Medicine* website (10.1038/s41746-018-0055-z).
