# Supplementary material for: Tracking health seeking behavior during an Ebola outbreak via mobile phones and SMS
Source: NPJ Digit Med. 2018 Oct 2;1:51. doi: 10.1038/s41746-018-0055-z (PMC6550280; doi:10.1038/s41746-018-0055-z)
Supplement: Supplementary file 1 — Supplementary information [file 41746_2018_55_MOESM1_ESM.pdf]

Supplementary Information
Tracking Health Seeking Behavior During an Ebola Outbreak via Mobile Phones and SMS
Feng et al.

Cellphone penetration in Liberia

Liberia has seen rapid growth of mobile phone penetration in recent years: in 2007 only 30% of Liberian households owned a mobile phone but this figured increased to 65% by the time of the 2013 Demographic and Health Survey (DHS)[1,2] and by the time of the GeoPoll survey in 2015 was anticipated to be much higher.

Survey Details

Round	Dates	Completion Rate (accounting for refusals and non-responses)
1	March 6-12 2015	10.7%
2	April 13-15 2015	18.1%
3	May 18-19 2015	18.8%
4	June 9-11, 2015	18.7%

Supplementary Table 1: Survey dates and completion rates

Survey Questions

Total of 10 questions were included, but only those relevant to the study are outlined below. Questions were translated into Liberian Pigeon according to the language preference of the user (determined by GeoPoll). For responses with options, the response would be via entering the corresponding number. Birth timing question options are as given in the final round of the survey, where May 2014 was 12 months prior. We experimented with different forms of the birth month response question (e.g. by reversing the months) to reduce answer bias due to response order.

Question	Options
What is your current occupational status?	1) Unemployed 2) Professional or technical or managerial 3) Sales and services 4) Laborer 5) Agriculture 6) Other
What is the highest level of school you have attended?	1) Primary school 2) Secondary school 3) Post-secondary school 4) No School
Are you male or female?	1) Male 2) Female
Have you given birth to a baby in the past year (since May 1, 2014)?	1) Yes 2) No
When was this baby born?	1) May 2014 2) Jun 2014 3) Jul 2014 4) Aug 2014 5) Sep 2014 6) Oct 2014 7) Nov 2014 8) Dec 2014 9) Jan 2014 10) Feb 2015 11) Mar 2015 12) Apr 2015 13) May 2015

Where was this baby born?	1) Government health facility or clinic 2) Private health facility or clinic 3) Your home or someone else's home 4) Other

Supplementary Table 2: Relevant survey questions and the options given

Costs per Survey

Incentives ($0.50 per survey), translation, survey and screening fees, plus one-time administration and translation fee for a total of $6 per survey.

Propensity score results

	GeoPoll (n=1009)	DHS (n=1009)	Standardized mean differences
**Age (%)**			0.123
18	41 (4.1)	39 (3.9)	
19	50 (5.0)	52 (5.2)	
20	55 (5.5)	62 (6.1)	
21	57 (5.6)	66 (6.5)	
22	76 (7.5)	59 (5.8)	
23	88 (8.7)	86 (8.5)	
24	42 (4.2)	39 (3.9)	
25	70 (6.9)	64 (6.3)	
26	65 (6.4)	57 (5.6)	
27	52 (5.2)	48 (4.8)	
28	48 (4.8)	46 (4.6)	
29	45 (4.5)	45 (4.5)	
30	39 (3.9)	45 (4.5)	
31	23 (2.3)	22 (2.2)	
32	28 (2.8)	29 (2.9)	
33	19 (1.9)	27 (2.7)	
34	16 (1.6)	22 (2.2)	
35	20 (2.0)	22 (2.2)	
36	34 (3.4)	28 (2.8)	
37	27 (2.7)	27 (2.7)	
38	22 (2.2)	20 (2.0)	
39	28 (2.8)	20 (2.0)	
40	21 (2.1)	23 (2.3)	
41	12 (1.2)	16 (1.6)	
42	7 (0.7)	10 (1.0)	
43	6 (0.6)	10 (1.0)	
44	4 (0.4)	3 (0.3)	
45	3 (0.3)	4 (0.4)	

46	3 (0.3)	7 (0.7)	
47	4 (0.4)	5 (0.5)	
48	0 (0.0)	4 (0.4)	
49	4 (0.4)	2 (0.2)	
**Occupation (%)**			0.050
Agriculture	178 (17.6)	198 (19.6)	
Laborer	59 (5.8)	67 (6.6)	
Other	35 (3.5)	41 (4.1)	
Professional	48 (4.8)	53 (5.3)	
Sales and services	185 (18.3)	178 (17.6)	
Unemployed	504 (50.0)	472 (46.8)	
**Region (%)**			0.106
Bomi	60 (5.9)	52 (5.2)	
Bong	102 (10.1)	111 (11.0)	
Gbarpolu	10 (1.0)	12 (1.2)	
Grand Bassa	46 (4.6)	53 (5.3)	
Grand Cape Mount	25 (2.5)	32 (3.2)	
Grand Gedeh	70 (6.9)	55 (5.5)	
Grand Kru	9 (0.9)	6 (0.6)	
Lofa	78 (7.7)	86 (8.5)	
Margibi	93 (9.2)	84 (8.3)	
Maryland	59 (5.8)	59 (5.8)	
Montserrado	244 (24.2)	261 (25.9)	
Nimba	165 (16.4)	144 (14.3)	
River Cess	6 (0.6)	8 (0.8)	
River Gee	11 (1.1)	14 (1.4)	
Sinoe	31 (3.1)	32 (3.2)	
**Education (%)**			0.069
No education	43 (4.3)	46 (4.6)	
Primary	256 (25.4)	267 (26.5)	
Secondary	626 (62.0)	603 (59.8)	
Higher	84 (8.3)	93 (9.2)	

Supplementary Table 3a: Standardized mean differences (SMD) in covariate values across treatment and comparison conditions the difference in the proportions/means across the treatment groups divided by the standard deviation within the treatment group[3] during the outbreak.

	GeoPoll (n=465)	DHS (n=465)	Standardized mean differences
**Age (%)**			0.231
18	29 (6.2)	24 (5.2)	
19	22 (4.7)	24 (5.2)	
20	24 (5.2)	26 (5.6)	
21	30 (6.5)	30 (6.5)	
22	23 (4.9)	27 (5.8)	
23	33 (7.1)	37 (8.0)	
24	19 (4.1)	20 (4.3)	
25	22 (4.7)	30 (6.5)	
26	31 (6.7)	30 (6.5)	
27	23 (4.9)	20 (4.3)	
28	25 (5.4)	20 (4.3)	
29	19 (4.1)	18 (3.9)	
30	30 (6.5)	23 (4.9)	
31	9 (1.9)	6 (1.3)	
32	15 (3.2)	14 (3.0)	
33	15 (3.2)	13 (2.8)	
34	10 (2.2)	11 (2.4)	
35	10 (2.2)	13 (2.8)	
36	14 (3.0)	17 (3.7)	
37	11 (2.4)	13 (2.8)	
38	11 (2.4)	9 (1.9)	
39	13 (2.8)	9 (1.9)	
40	4 (0.9)	5 (1.1)	
41	5 (1.1)	5 (1.1)	
42	4 (0.9)	3 (0.6)	
43	6 (1.3)	3 (0.6)	
44	3 (0.6)	2 (0.4)	
45	1 (0.2)	3 (0.6)	
46	2 (0.4)	4 (0.9)	
47	1 (0.2)	2 (0.4)	
48	0 (0.0)	1 (0.2)	
49	1 (0.2)	3 (0.6)	
**Occupation (%)**			0.075
Agriculture	81 (17.4)	78 (16.8)	
Laborer	39 (8.4)	33 (7.1)	
Other	30 (6.5)	31 (6.7)	
Professional	31 (6.7)	38 (8.2)	
Sales and services	84 (18.1)	86 (18.5)	
Unemployed	200 (43.0)	199 (42.8)	

**Region (%)**			0.170
Bomi	11 (2.4)	17 (3.7)	
Bong	57 (12.3)	61 (13.1)	
Gbarpolu	9 (1.9)	10 (2.2)	
Grand Bassa	23 (4.9)	25 (5.4)	
Grand Cape Mount	9 (1.9)	14 (3.0)	
Grand Gedeh	20 (4.3)	24 (5.2)	
Grand Kru	2 (0.4)	2 (0.4)	
Lofa	49 (10.5)	38 (8.2)	
Margibi	35 (7.5)	41 (8.8)	
Maryland	25 (5.4)	23 (4.9)	
Montserrado	124 (26.7)	120 (25.8)	
Nimba	80 (17.2)	67 (14.4)	
River Cess	2 (0.4)	2 (0.4)	
River Gee	10 (2.2)	9 (1.9)	
Sinoe	9 (1.9)	12 (2.6)	
**Education (%)**			0.101
No education	14 (3.0)	18 (3.9)	
Primary	99 (21.3)	98 (21.1)	
Secondary	269 (57.8)	251 (54.0)	
Higher	83 (17.8)	98 (21.1)	

Supplementary Table 3b: Standardized mean differences (SMD) in covariate values across treatment and comparison conditions the difference in the proportions/means across the treatment groups divided by the standard deviation within the treatment group[3] for post outbreak peak.

Supplementary Figure 1 Legend: Distribution of propensity scores for treatment (GP) and control (DHS) groups during the outbreak (a and c), and post outbreak peak (b and d), prior to and after the match.

References

1 Demographic and Health Surveys. Liberia - Key Findings. (2007).

2 Demographic and Health Surveys. Liberia - Key Findings. (2013).

3 Stuart, E. A. Matching methods for causal inference: A review and a look forward. *Statistical science: a review journal of the Institute of Mathematical Statistics* **25**, 1 (2010).
