# Supplementary material for: Tracking health seeking behavior during an Ebola outbreak via mobile phones and SMS
Source: NPJ Digit Med. 2018 Oct 2;1:51. doi: 10.1038/s41746-018-0055-z (PMC6550280; doi:10.1038/s41746-018-0055-z)
Supplement: Supplementary file 2 — Supplemental Figure 1 [file 41746_2018_55_MOESM2_ESM.pdf]

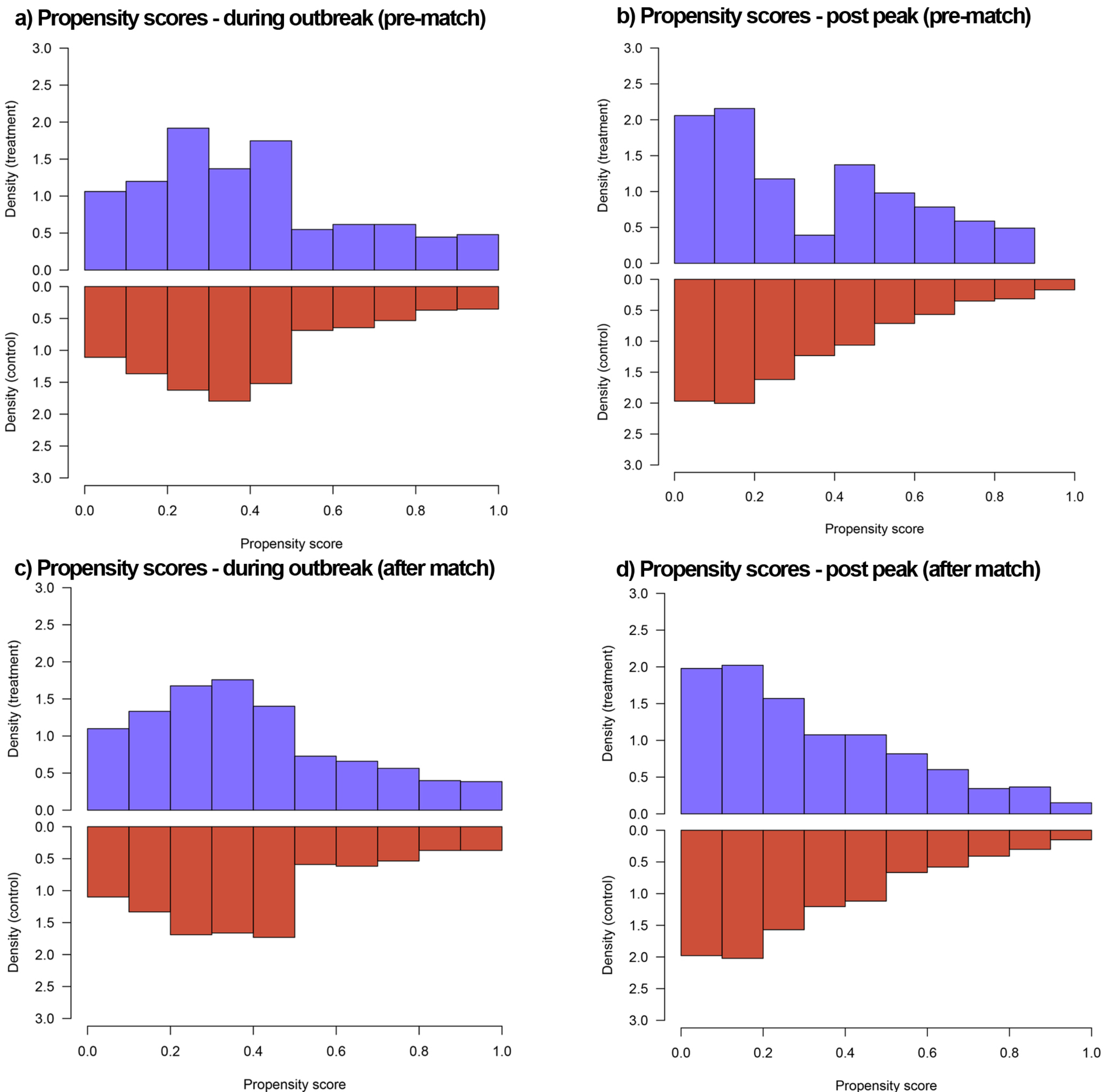
a) Propensity scores - during outbreak (pre-match)
Density (treatment)
Density (control)
Propensity score
b) Propensity scores - post peak (pre-match)
Density (treatment)
Density (control)
Propensity score
c) Propensity scores - during outbreak (after match)
Density (treatment)
Density (control)
Propensity score
d) Propensity scores - post peak (after match)
Density (treatment)
Density (control)
Propensity score
0.0
0.5
1.0
1.5
2.0
2.5
3.0
0.2
0.4
0.6
0.8
